# Supplementary material for: Three-dimensional digital mapping of ecosystems: a new era in spatial ecology
Source: Proc Biol Sci. 2020 Feb 12;287(1920):20192383. doi: 10.1098/rspb.2019.2383 (PMC7031661; doi:10.1098/rspb.2019.2383)
Supplement: Detailed methods and supplementary results [file rspb20192383supp1.pdf]

Proceedings of the Royal Society B

DOI: 10.1098/rspb.2019.2383

Three-dimensional digital mapping of ecosystems: a new era in spatial ecology

Tim D'Urban Jackson[1], Gareth J. Williams[1], Guy Walker-Springett[1] and Andrew J. Davies[1, 2]

[1]School of Ocean Sciences, Bangor University, Anglesey, LL59 5AB, UK

[2]Department of Biological Sciences, University of Rhode Island, Kingston, RI, USA

Supplementary material, S1

Methods for an accuracy assessment of structure-from-motion photogrammetry models for 3D mapping in ecological contexts.

Error in structure-from-motion photogrammetry models was tested by comparison with terrestrial laser scanner data of plots in three common and ecologically important habitats (rocky shore, saltmarsh and honeycomb worm reef) that together cover approximately 72% of UK intertidal land [1]. Rocky shores are a classic model for investigating relationships between biodiversity and habitat structural complexity [2]. Saltmarshes are vegetated habitats with both terrestrial and marine features where fine-scale variation in topography can result in substantial biological and physical responses [3]. Honeycomb worm (*Sabellaria alveolata*) reef is a habitat of conservation importance listed in national and international environmental legislation, making up the most significant intertidal bioconstructions in Europe [4]. Study sites were located along the North Wales coast (UK) with fieldwork conducted on spring low tides during summer and autumn 2017.

Terrestrial laser scanning and structure-from-motion surveys for each plot were conducted simultaneously for direct comparison of outputs. Weather conditions were optimal for both survey techniques, with data collected on days with sunshine, low wind speed and no precipitation. Three spatial scales were tested to maximise the relevance of results to a wide range of ecological study designs: three 25 m^2 quadrats per habitat with a target spatial resolution of < 1 cm (fine-scale), a single 2500 m^2 area per habitat with < 2 cm resolution (medium-scale) and the same 2500 m^2 area with 5 cm resolution (broad-scale). Fine-scale plots represent quadrat scale field sampling, medium-scale plots tested the level of detail and accuracy achievable at a habitat scale and broad-scale sampling was based on a typical design for a large extent drone survey that could be used at ecosystem scales.

The same tripod-mounted terrestrial laser scanning equipment (Leica Geosystems ScanStation C10) was used at all scales. All surveys used full field-of-view (360° horizontal, 270° vertical), medium resolution scans (point spacing of 10 cm at 100 m range) with no photographs. Fine-scale plots used four scanning stations while medium- and broad-scale plots used 7–8 stations. 3D mapping using structure-from-motion requires simply a set of overlapping photographs of a scene. Photographs for SfM were taken using a pole mounted camera (18 MP Canon EOS M with 22mm prime lens) for fine scale plots, and a quadrocopter drone (DJI Phantom 3 Pro with 12 MP camera) for medium- and broad-scale plots, flying at 25 m and 90 m altitude respectively. The drone was flown on an automated parallel track flight path by a professional drone pilot. Shared reference targets were used for terrestrial laser scanning and structure-from-motion so data from the two techniques could be aligned without georeferencing, minimising error introduction. Fine-scale plots included scaling objects which provided scale reference along x, y and z axes. Medium- and broad-scale plots included reference objects of known size and shape for comparison of results.

After data collection, both terrestrial laser scanning and structure-from-motion require a series of data processing steps to ensure high quality outputs. With calibrated equipment, data from each terrestrial laser scanning station are correctly scaled, levelled and have known precision (6 mm individual measurement precision at a range of 50 m for the equipment we used). Data processing was conducted using Leica Geosystems Cyclone and involves aligning data from multiple stations using reference target positions or regions of overlapping geometry. Station data were aligned to a single complete dataset so that 3D errors in target positions between individual stations were a maximum of 6 mm. The complete point cloud dataset was manually cleaned of unwanted objects (e.g. people), artefacts (e.g. reflections in water surfaces) and noise (erroneous points).

Data processing for structure from motion was conducted using the popular software Agisoft Photoscan Professional. This software option was chosen because it provides a good balance between quality of outputs, control over settings, user-friendliness and cost. The workflow for processing structure-from-motion data using Photoscan is similar to other software options. First, images were

checked for sharpness and exposure, blurred images were discarded and exposure was corrected where necessary. Background and moving features like shadows were masked from images. Images were automatically aligned using “High” accuracy and 40,000 tie points to generate a sparse point cloud. Markers were manually placed at the centre of each reference target in each image. For fine-scale image sets, 15-19 pairs of scale markers were placed at various separations from 1 cm to 1 m. Shared reference markers were assigned the same coordinates as in the relevant terrestrial laser scanning dataset. Image alignment was iteratively optimised after marker placements and using the gradual selection tool to delete low quality tie points using a workflow adapted from [5]. A dense cloud was then generated using “High” quality setting and “Mild” depth filtering to retain fine topographic features while removing noise. The resulting point cloud was exported for analysis.

Pairs of aligned terrestrial laser scanning and structure-from-motion point cloud models were cropped to common extents, subsampled to similar point densities and further cleaned using the statistical outlier removal tool in the open source software CloudCompare. Broad-scale terrestrial laser scanning data were generated by subsampling medium-scale data to a density similar to broad-scale drone data (5 cm point spacing).

The distance between each pair of models was measured at 100,000 random positions using the multiscale model-to-model cloud comparison algorithm (M3C2), a robust method developed specifically for comparison of point cloud data from natural environments which contain multiscale complexity, implemented in the open source software CloudCompare [6]. In brief, the algorithm calculates the distance between point clouds in the direction of the local surface orientation at each measured point. This is an improvement over nearest neighbour methods or measuring distance along a single axis, typically vertically.

The mean of absolute distances measured was used to quantify the accuracy of the structure-from-motion model relative to the terrestrial laser scanning model, as mean absolute error (MAE) (figure 3). While another metric, root mean square error (RMSE) is commonly used for model comparisons,

this can be heavily influenced by a small number of large errors which were likely to be present in our data due to some noise remaining after data cleaning. We visually analysed point cloud models and cross sections to determine the key differences between outputs from two different techniques (electronic supplementary material, figure S2).

References

1. Rowland C, Morton D, Carrascao Tornero L, McShane G, O'Neil A, Wood C. 2017 Land Cover Map 2015 (25 m raster, GB).

2. Kovalenko KE, Thomaz SM, Warfe DM. 2012 Habitat complexity: Approaches and future directions. *Hydrobiologia* **685**, 1–17. (doi:10.1007/s10750-011-0974-z)

3. Langlois E, Bonis A, Bouzillé J. 2003 Sediment and plant dynamics in saltmarshes pioneer zone: Puccinellia maritima as a key species? *Estuar. Coast. Shelf Sci.* **56**, 239–249. (doi:10.1016/S0272-7714(02)00185-3)

4. Desroy N, Dubois SF, Fournier J, Ricquiers L, Le Mao P, Guerin L, Gerla D, Rougerie M, Legendre A. 2011 The conservation status of Sabellaria alveolata (L.) (Polychaeta: Sabellariidae) reefs in the bay of mont-saint-michel. *Aquat. Conserv. Mar. Freshw. Ecosyst.* **21**, 462–471. (doi:10.1002/aqc.1206)

5. Mayer C, Gomes Pererira LM, Kersten TP. 2018 A Comprehensive Workflow to Process UAV Images for the Efficient Production of Accurate Geo-information. In *CNCG2018 - IX Conferência Nacional de Cartografia e Geodesia, October 25-26, 2018, At Amadora, Portugal,*

6. Lague D, Brodu N, Leroux J. 2013 Accurate 3D comparison of complex topography with terrestrial laser scanner: Application to the Rangitikei canyon (N-Z). *ISPRS J. Photogramm. Remote Sens.* **82**, 10–26. (doi:10.1016/j.isprsjprs.2013.04.009)

Supplementary material, figure S2

Visual examples of differences between terrestrial laser scanning and structure-from-motion derived 3D habitat models

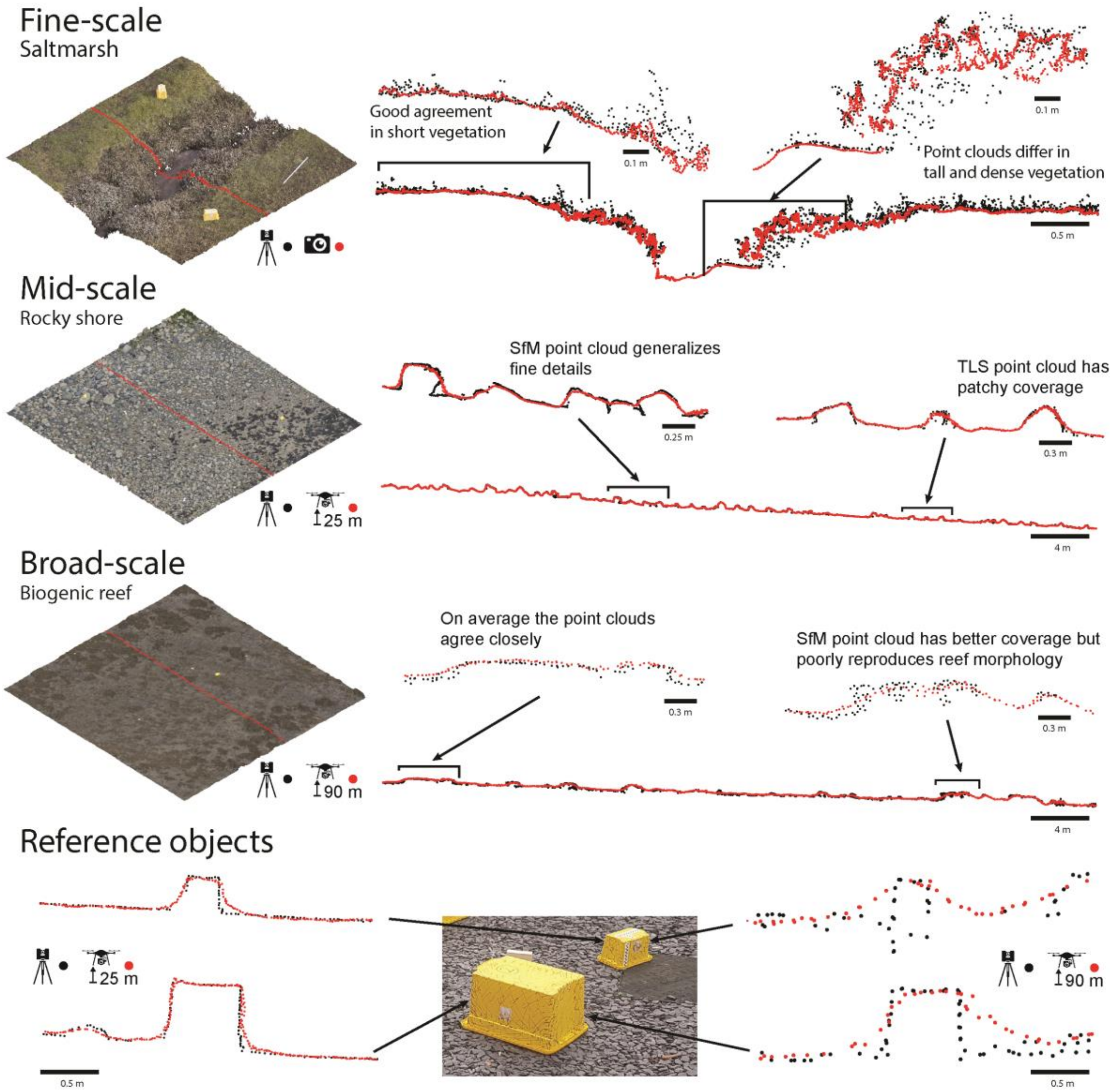

Differences in 3D point cloud models generated by terrestrial laser scanning (TLS, black points) and structure-from-motion photogrammetry (SfM, red points) at three spatial scales and three habitats are demonstrated. Models agree closely at fine-scales (25 m^2 extent, <1 cm resolution) in areas of solid substrate or short vegetation. In tall and dense vegetation the models differ, with points captured from

further in to the feature by terrestrial laser scanning. At medium-scales (2500 m^2 extent, <2 cm resolution) on solid substrate average difference in models is low, but fine details are generalised by structure-from-motion. Terrestrial laser scanning data have gaps due to some areas being out of line-of-sight from any scanning position. At broad-scales (2500 m^2 extent, 5 cm resolution) SfM models the general form of the scene well but detailed topographic morphology is more accurate in terrestrial laser scanner data. As scale increases detailed features become smoothed by structure-from-motion, as demonstrated by models of reference objects with known shape and size.
